# Beneficial Effects of Catalpol Supplementation during In Vitro Maturation of Porcine Cumulus-Oocyte Complexes

**DOI:** 10.3390/antiox12061222

**Published:** 2023-06-05

**Authors:** Yanxin Wang, Ye Xu, Sijia Li, Xi Yan, Xiaofen Yang, Mengjia Chen, Yun Wang, Ruru Jia, Dongping Zhou, Deshun Shi, Fenghua Lu

**Affiliations:** 1 State Key Laboratory for Conservation and Utilization of Subtropical Agro-Bioresources, Guangxi University, 75 Xiuling Road, Nanning 530005, China; abbywangsir@163.com (Y.W.); 2018391048@st.gxu.edu.cn (Y.X.);; 2 Guangxi Key Laboratory of Animal Breeding, Disease Control and Prevention, Guangxi University, 75 Xiuling Road, Nanning 530005, China

**Keywords:** oocytes, in vitro maturation, catalpol, antioxidant

## Abstract

Oxidative stress degrades oocytes during in vitro maturation (IVM). Catalpol, a well-known iridoid glycoside, exhibits antioxidant, anti-inflammatory, and antihyperglycemic effects. In this study, catalpol supplementation was tested on porcine oocyte IVM and its mechanisms. Corticalgranule (GC) distribution, mitochondrial function, antioxidant capacity, DNA damage degree, and real-time quantitative polymerase chain reaction were used to confirm the effects of 10 μmol/L catalpol in the maturation medium during IVM. Catalpol treatment significantly increased the first-pole rate and cytoplasmic maturation in mature oocytes. It also increased oocyte glutathione (GSH), mitochondrial membrane potential and blastocyst cell number. However, DNA damage as well as reactive oxygen species (ROS) and malondialdehyde (MDA) levels. Mitochondrial membrane potential and blastocyst cell number were also increased. Thus, the supplementation of 10 μmol/L catalpol in the IVM medium improves porcine oocyte maturation and embryonic development.

## 1. Introduction

Oocyte in vitro maturation (IVM) is a process of oocyte meiosis, in an in vitro environment, from the first meiotic prophase (germinal vesicle stage) to the second meiotic intermediate phase (metaphase II (MII) stage). Oocyte IVM is a key step in in vitro embryo production and plays a significant role in fertilization and subsequent embryonic development [1,2]. IVM can provide a source of oocytes for embryo production and transfer, animal cloning, transgenic animal production, and human ovarian xenotransplantation.

Currently, a large gap between in vivo and in vitro maturation of oocytes exists, due to developmental arrest caused by an unstable IVM environment (oxidative stress, and suboptimal temperature, pH, and artificial culture fluid composition) [3]. Oxidative stress, which has the largest negative impact on embryonic development, results from the failure of the antioxidant defense system, leading to the accumulation of reactive oxygen species (ROS) [4]. Low-to-moderate doses of ROS are essential for regulating normal physiological functions, the immune system, and redox homeostasis [5,6]; however, excessive cellular ROS levels cause damage to lipids, nucleic acids, proteins, membranes, and organelles (such as mitochondria), which can lead to the activation of cell death [7,8,9]. It has been shown that the levels of ROS and antioxidants in the IVM medium affect oocyte maturation and blastocyst formation [10,11]. Excessive amounts of ROS have deleterious effects on IVM oocytes by the induction of organelle dysfunction, spindle abnormality, DNA fragmentation, and apoptosis [12,13]. The addition of antioxidants to the culture medium protects oocytes from oxidative stress and improves mature oocyte culture efficiency and quality, which is crucial for in vitro embryo production [14].

Catalpol, a highly active cyclic enol ether terpenoid, is primarily extracted from groundnuts and has anti-inflammatory, anti-apoptotic, hypoglycemic, antiviral, and antioxidant effects [15]. In diabetic rabbits, catalpol has been shown to significantly reduce serum ROS and malondialdehyde (MDA) levels and increase superoxide dismutase (SOD) activity and total antioxidant capacity [16]. During IVM, numerous antioxidant substances are used to maintain intracellular redox homeostasis and improve oocyte quality [17,18,19]. However, little is known about the effects of catalpol on the in vitro or germ cell line maturation of porcine oocytes. Catalpol is a strong antioxidant [20,21,22]; therefore, we hypothesized that catalpol supplementation will positively impact IVM rate and porcine oocyte quality.

The main aim of this study was to investigate the effect of catalpol on porcine cumulus-oocyte complex (COCs) IVM, and subsequent embryonic developmental competence in parthenogenetic activation (PA).

## 2. Materials and Methods

### 2.1. Chemicals and Reagents

Catalpol was purchased from the Chengdu Manstead (Manster Biotechnology Co., Chengdu, China) and dissolved in tissue culture medium 199 (TCM199). The catalpol-supplemented medium was stored at −20 °C. TCM199 and fetal bovine serum (FBS) were obtained from Gibco (Grand Island, NY, USA). The ROS, glutathione (GSH), and mitochondrial membrane potential assay kits (S0033, S0052, and C2003S, respectively) were purchased from Beyotime Biotechnology Inc. (Shanghai, China). Unless otherwise stated, all chemicals and reagents used in this study were purchased from Sigma-Aldrich (St. Louis, MO, USA).

### 2.2. IVM of Porcine COCs

Porcine ovaries were obtained from a nearby slaughterhouse (Shuichang Road Slaughterhouse, Nanning, China), deposited in a saline solution pre-heated to 37 °C, and immediately transported to the laboratory. Alcohol and saline were used to clean the ovaries three times each. COCs were extracted from 2 to 6 mm follicles. We selected COCs with a homogeneous cytoplasm and three or more layers of compact cumulus cells (CCs), using a stereomicroscope (SMZ645; Nikon, Tokyo, Japan). Selected COCs were washed two to three times with 2% FBS-supplemented TCM199. Finally, the COCs were incubated in a maturation medium (a modified bicarbonate-buffered TCM-199 medium) for 44 h at 38.5 °C, 5% CO_2_, and 100% humidity. Catalpol (0, 5, 10, 50, and 100 mol/L) was added to the maturation medium at different amounts. The extrusion of the first polar body after IVM was used to evaluate oocyte maturity under a stereomicroscope.

### 2.3. PA of Porcine Oocytes

After IVM, COCs were placed in a 0.1% hyaluronidase solution. Oocytes were observed under a microscope and after gentle blowing with a 100 μL pipette, oocytes with intact zona pellucida, normal cytoplasm, and discharged first polar body were collected. Mature oocytes were placed in ionomycin for 5 min, washed two to three times with NCSU-23, and transferred to an N6-dimethyladenine incubator for 3.5 to 4 h. Ten to 20 oocytes were transferred into 35 μL NCSU-23 drops contained on culture plates covered with 3 mL paraffin oil The plates were incubated at 38.5 °C and 5% CO_2_. The oocyte cleavage and number of blastocysts were measured at 48 h and 168 h, respectively.

### 2.4. cDNA Acquisition from MII Stage Porcine Oocytes

Seven MII-stage oocytes were washed three times with phosphate-buffered saline (PBS) and placed in a 200 μL centrifuge tube containing 10 μL lysate. The tubes were placed in a polymerase chain reaction (PCR) instrument for 15 min at 75 °C. Subsequently, 1 μL DNase I (Fermentas) (1 IU) and 1.3 μL 10×RDP buffer was added and the centrifuge tubes were placed in a PCR instrument for 30 min at 37 °C. Then, 1 μL EDTA (50 mM), 4 μM random primer (TaKaRa Bio, Kusatsu, Shiga, Japan), and 1 μL mixture deoxynucleotides (dNTPs, (TaKaRa Bio; 10 mM) were added and the centrifuge tubes were placed in a PCR instrument for 10 min at 65 °C. Finally, 4 μL 5×First-Strand Buffer, 2 μL dithiothreitols (0.1 M), 0.25 μL SuperScriptTM II Reverse Transcriptase (Invitrogen, Waltham, MA, USA), and 0.5 μL RNase Inhibitor (TaKaRa Bio) was added and the centrifuge tubes were placed in a PCR instrument for 10 min at 25 °C, 90 min at 42 °C, 10 min at 95 °C, and cooled to 4 °C. The procedures were performed on ice.

### 2.5. RNA Isolation and Real-Time Quantitative PCR

Extraction of CC RNA was performed using the TRIZOL reagent (Vazyme Biotech Co., Nanjing, China), according to the manufacturer’s instructions. Using the PrimeScript™ reagent kit (TaKaRa, Bio), 500 ng of the template was reverse transcribed into cDNA. The SYBR green assay system was used for quantitative real-time PCR (qRT-PCR) on an ABI 7500 Fast RT-PCR System (Applied Biosystems, Waltham, MA, USA). Table 1 lists the primers used for qRT-PCR. One cycles at 95 °C for 30 s, 40 cycles at 95 °C for 5 s and 60 °C for 45 s were the reaction conditions. Threshold cycle-based relative measurement was conducted at constant fluorescence intensity. Calculating relative mRNA expression (R):

$$R=2^{-[\Delta Ct\;sample-\Delta Ct\;control]}$$

CCs and MII-stage oocytes normalized each mRNA to β-actin.

### 2.6. Evaluation of CC Expansion

During IVM, different concentrations of catalpol (0, 5, 10, 50, and 100 mol/L) were utilized, and CC expansion was measured. CC expansion was calculated using a previously published rating method (grade 1–3) [23]. Grade 1 showed no CC expansion and was worth one point. Grade 2 is the small expansion of numerous layers of CCs, but cell-to-cell contact remained very tight; clustered cells were still detected, and this was worth two points. Grade 3 represented the entire or almost complete expansion of CCs and was worth three points.

### 2.7. Examination of Mitochondrial Membrane Potential in Oocytes

Mitochondrial membrane potential was examined using a mitochondrial membrane Potential Kit (Beyotime Biotechnology, Inc.), according to the manufacturer’s instructions. Briefly, denuded oocytes (DOs) were cultured with 0.05% JC-1 fluorescent dye for 30 min at 38.5 °C. After washing several times with 0.1% buffered saline—Professional Video Assistant phosphate (PBS-PVA), images were captured using a fluorescence microscope (EVOS FL Auto, Thermo Fisher, Waltham, MA, USA). The fluorescence intensity of the oocytes was calculated using ImageJ-win64 software (National Institutes of Health).

### 2.8. Examination of Mitochondrial Distribution in Oocytes

Oocytes were incubated in PBS with 3% BSA and 180 nM Mito Tracker Red CMX Ros (Beyotime Biotechnology) for 30 min at 38.5 °C and 5% CO_2_. Oocytes were placed on glass slides and imaged using a confocal microscope (Leica, Wetzlar, Germany) after repeated washes with 0.1% PBS-PVA. ImageJ computed oocyte fluorescence intensity (National Institutes of Health).

### 2.9. ROS Assay

Intracellular oocyte ROS levels were measured using a ROS test kit (Beyotime Biotechnology Inc.). MII stage oocytes were treated with 10 μmol/L dichlorofluorescein diace-tate (DCFH-DA; fluorescent probe) for 30 min at 38.5 °C. After three washes with 0.1% PBS-PVA, pictures were taken with a fluorescence microscope (EVOS FL Auto) at constant settings. The mean optical density values (IntDen/Area) were analyzed using ImageJ software (National Institutes of Health).

### 2.10. Total GSH Determination

The oocytes were washed three times with PBS-PVA, and three times the volume of the protein removal reagent S. The oocytes were freeze-thawed three times in liquid nitrogen and a 37 °C water bath, placed on ice for 5–10 min, and centrifuged at 10,000× *g* for 10 min at 4 °C, collecting supernatant. According to the manufacturer’s instructions, the total GSH was determined using the total GSH assay kit (Beyotime Biotechnology Inc.). Working and 0.5 mg/mL nicotinamide adenine dinucleotide phosphate (NADPH) solutions were prepared. For standard curve preparation, 25, 15, 10, 5, and 2 μM GSH solutions were prepared using protein removal reagent S. Total GSH assay working solution (150 μL) was mixed with 10 μL of the collected supernatant or standard in a 96-well plate. The plate was incubated for 5 min at room temperature (25 °C), 50 μL of 0.5 mg/mL NADPH solution was added, and the solution was mixed well. After 25 min, the absorbance was measured at 412 nm, using an enzyme marker (Tecan M200 PRO, Männedorf, Switzerland).

### 2.11. Malondialdehyde (MDA) Assay

The oocytes around the MIIstage were blown clean, and 50 oocytes from each group were collected, washed three times with PBS-PVA, lysed on ice with western and immunoprecipitation cell lysis solution (P0013), and centrifuged at 10,000–12,000× *g* for 10 min to remove the supernatant for analysis. The thiobarbituric acid (TBA) storage solution and MDA assay working solution were prepared according to the lipid oxidation assay kit (Beyotime Biotechnology Inc.) instructions (ratio of TBA dilution: TBA storage solution: antioxidant = 150:50:3). Lysate (100 μL) was used as a blank control and 1, 2, 5, 10, 20, 50 μM dilutions were prepared with distilled water, for the standard curve. The sample (100 μL) and 200 μL of the MDA assay working solution were added to the EP tube, mixed, heated in a boiling water bath for 15 min, cooled to room temperature (25 °C), and centrifuged at 1000× *g* for 10 min. The supernatant (200 μL) was added to a 96-well plate and quantified at 532 nm with an enzyme marker. To prevent leakage of the boiling liquid, the EP tubes were coated with a sealing film and kept away from the heating surfaces.

### 2.12. Detection of CG Distribution in Porcine Oocytes

The control and optimal catalpol concentration treatment groups were selected for subsequent experiments. Mature oocytes were incubated in 4% paraformaldehyde (PFA) for at least 30 min and washed three times with PBS-PVA. The oocytes were permeabilized overnight in EP tubes containing 1% Triton X-100, washed three times with BSA, and incubated overnight at 4 °C in peanut lectin lectin-FTTC (1:100). The tubes were washed three times with PBS-PVA under light-proof conditions, pressed, and photographed using a laser confocal microscope.

### 2.13. Immunofluorescence Staining

CCs around porcine oocytes were removed after maturation using 0.1% hyaluronidase digestion and 200 μL pipette blowing. Next, 30-min PBS-PVA washes in 4% PFA, were performed three times. Oocytes were extracted and rinsed three times with a blocking solution for 5 min, to complete fixation. Blocking solution washes were performed for 5 min and the oocytes were fully permeabilized for 30 min at room temperature (25 °C) using 1% Triton X-100. The oocytes were closed with 1% BSA for 1 h and washed three times with PBS for 5 min. Next, oocytes were incubated in 1% BSA-diluted primary antibody overnight at 4 °C. Oocytes were washed three times with PBS at room temperature (25 °C). The oocytes were then incubated for 1.5 h at room temperature (25 °C) with a 1% BSA-diluted secondary antibody before being rinsed three times with PBS for 5 min. Hoechst 33,342 oocyte-staining was performed for 15 min. Three PBS-PVA washes were performed for 5-min. Oocytes were sealed with petroleum jelly on clean slides and photographed under a confocal microscope.

### 2.14. Blastocyst Hoechst33342 Staining

After PA, we collected the 7-day-old blastocysts and, washed them three times with PBS-PVA. Following this, they were placed in 200 μL EP tubes containing 4% PFA and incubated for 30 min. The stained blastocysts were washed three times with PBS-PVA, transferred to 10 μg/mL Hoechst 33,342 dye, and incubated in the dark for 15 min at 38.5 °C. The stained blastocysts were washed three times, placed on slides, sealed with petroleum jelly, and photographed under a fluorescence microscope. Finally, the ImageJ program tallied cell counts. (National Institutes of Health).

### 2.15. Statistical Analysis

All experiments were tripled. IBM SPSS Statistics 18.0 (IBM Corp., Armonk, NY, USA) was used for statistical analyses. To evaluate CC expansion, COC excretion rate, embryonic development, and relative gene expression levels, the one-way analysis of variance (ANOVA) and Duncan’s multiple range test were utilized. Data are presented as mean ± SEM. *p* < 0.05, *p* < 0.01, and *p* < 0.001 indicated statistical significance.

## 3. Results

### 3.1. Effects of Catalpol on Extrusion of the First Polar Body

Different catalpol concentrations (0, 5, 10, 50, and 100 µmol/L) were used during IVM and the effects were estimated using the first polar body rate (Table 2). The first polar body rate in the 10 μmol/L catalpol group (72.33 ± 1.93%) was significantly higher than that of the control group (61.07 ± 2.80%).

### 3.2. Effects of Catalpol on CC Expansion in Porcine COCs

**Figure 1:**
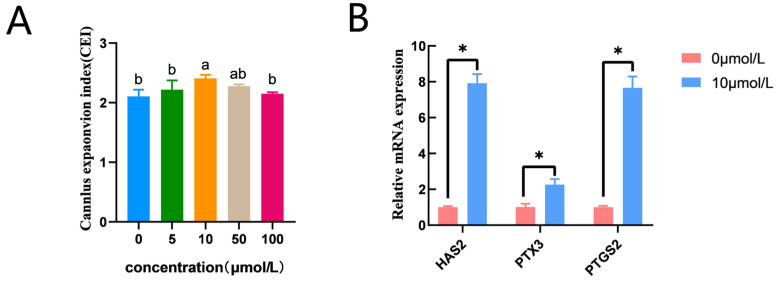
Effects of Catalpol on CC Expansion in porcine COCs. (**A**) cumulus expansion index (**B**) Cumulus expansion-related genes (*HAS2*, *PTX3* and *PTGS2*) were expressed in CCs treated with Catalpol for 44 h of IVM. The mRNA levels were normalized to β-actin expression as a control. The use of an asterisk denotes statistical significance (* *p* < 0.05). a/b values in the same column with different superscripts significantly differ (*p* < 0.05).

CC expansion in the 10 μmol/L catalpol treatment group (2.41 ± 0.07, n = 145) was significantly higher than that in the control group (2.11 ± 0.03, n = 150). (Figure 1A). In addition, prostaglandin endoperoxide synthase 2 (*PTGS2)*, pentraxin 3 (*PTX3)*, and hyaluronan synthase 2 (*HAS2*) mRNA expression was significantly higher in the catalpol group (Figure 1B).

### 3.3. Effects of Catalpol Supplementation on the Distribution of CGs inPorcine MII Stage Oocytes

**Figure 2:**
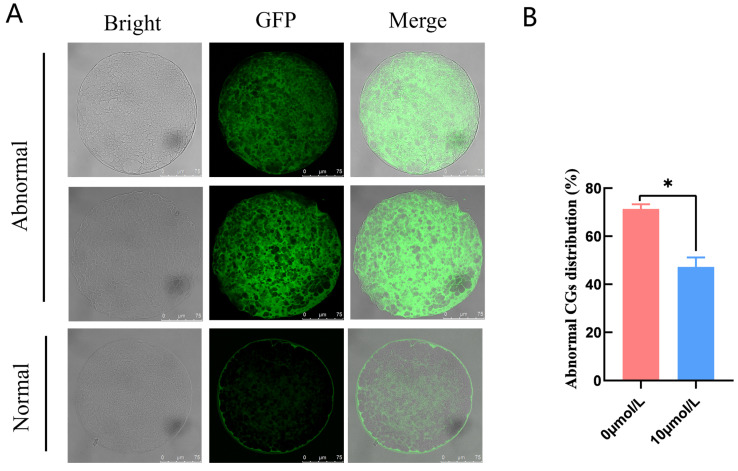
Effects of Catalpol on CGs in porcine MII stage oocytes. (**A**) stained fluorescent picture with LCA-FITC; (**B**) statistics of staining fluorescence intensity with LCA-FITC. Asterisks indicate statistical significance (* p < 0.05). scale bar = 75 μm.

Abnormal CG distribution in the 0 μmol/L catalpol treatment group (71.37 ± 1.93, n = 40) was significantly higher than in the 10 μmol/L catalpol treatment group (47.22 ± 3.93, n = 55) (Figure 2).

### 3.4. Effects of Catalpol on Mitochondrial Activity and Function in Porcine MII Stage Oocytes

**Figure 3:**
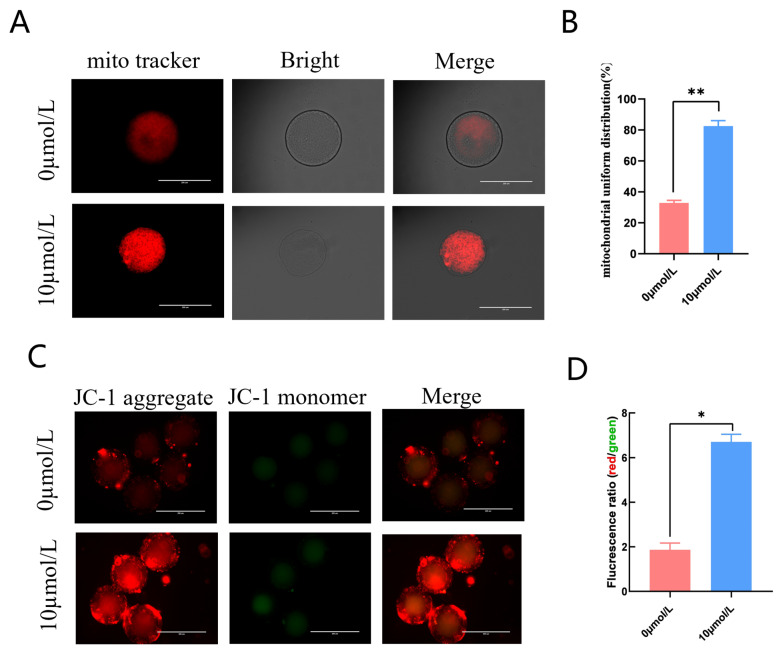
Effects of Catalpol on mitochondrial activity and function in porcine MII stage oocytes. (**A**) stained fluorescent picture with mito tracker; (**B**) mitochondrial uniform distribution in porcine oocytes; (**C**) stained fluorescent picture with JC-1; (**D**) statistics of staining fluorescence intensity with JC-1. The mean ± SEM was used to represent all data. Asterisks indicate statistical significance (* p < 0.05, ** p < 0.01). scale bar = 200 μm.

The 10 μmol/L catalpol treatment group had clustered, homogenous mitochondria (Figure 3A); in addition, this group had significantly more mitochondrial homogeneous dispersion than the 0 mol/L group. (85.20 ± 3.54, n = 46 vs. 32.91 ± 1.54, n = 54, *p* < 0.01; Figure 3B). We measured mitochondrial membrane potential using the fluorescent probe JC-1. The red/green JC-1 signal ratio in the 10 μmol/L catalpol treatment group was significantly higher than that of the control group (6.71 ± 0.34, n = 90 vs. 1.87 ± 0.31, n = 90, *p* < 0.05; Figure 3C,D), indicating increased porcine oocyte mitochondrial membrane potential.

### 3.5. Effects of Catalpol on Antioxidant Capacity in Porcine MII Stage Oocytes

**Figure 4:**
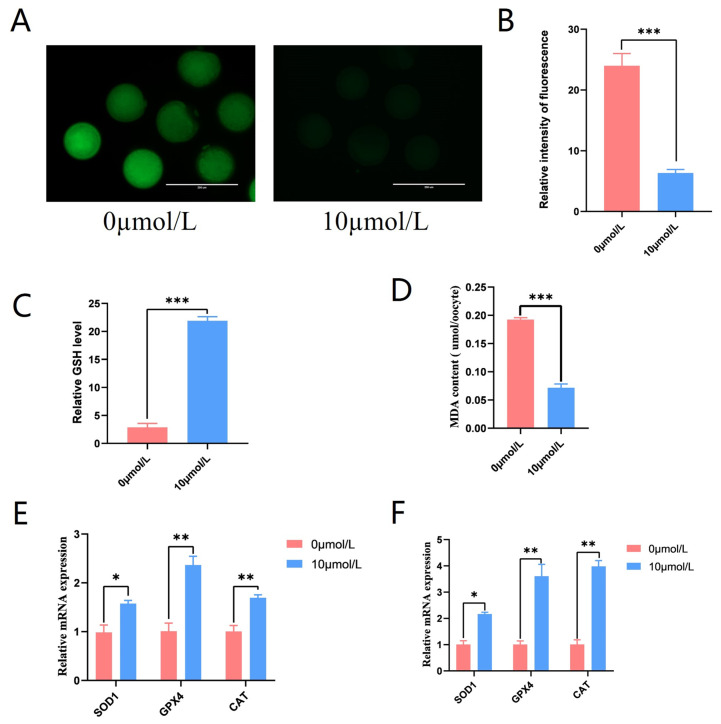
Effects of Catalpol on Antioxidant capacity in Porcine MII stage oocytes. (**A**) stained fluorescent picture with DCFH-DA; (**B**) statistics of staining fluorescence intensity with DCFH-DA; (**C**) Total glutathione content testing in porcine MII stage oocytes. (**D**) Malondialdehyde content testing in porcine MII stage oocytes. (**E**) Expression of Anti-oxidation-related genes (*SOD1*, *GPX4* and *CAT*) in CCs treated with catalpol during 44 h of IVM. (**F**) Expression of Anti-oxidation-related genes (*SOD1*, *GPX4* and *CAT*) in porcine MII stage oocytes treated with catalpol during 44 h of IVM. The mRNA levels were normalized to *β-actin* expression as a control. The mean ± SEM was used to represent all data. Asterisks indicate statistical significance (* *p* < 0.05, ** *p <* 0.01, *** *p* < 0.001). scale bar = 200 μm.

To explore the role of catalpol on ROS production in porcine oocytes, the fluorescent probe DCFH-DA was introduced into oocytes. The 10 μmol/L catalpol treatment group showed considerably lower ROS fluorescence intensity compared to the control group (6.33 ± 0.58, n = 90 vs. 24.00 ± 2.00, n = 90, *p* < 0.001; Figure 4A,B), and GSH levels were significantly higher in the 10 μmol/L catalpol treatment group compared to those in the control group (21.902 ± 0.74 vs. 2.88 ± 0.70, *p* < 0.001; Figure 4C). Furthermore, significantly lower MDA levels were observed in the 10 μmol/L catalpol treatment group compared to those in the control group (0.19 ± 0.04 vs. 0.07 ± 0.07, *p* < 0.001; Figure 4D). After porcine COCs were treated with catalpol, the mRNA expression of antioxidant genes significantly increased in CCs and MII-stage oocytes. These genes included superoxide dismutase1 (*SOD1*), catalase from Micrococcus lysodeikticus (*CAT*), and GSH peroxidase 1 (*GPX1*).

### 3.6. Effects of Catalpol on DNA Damage in Porcine MII Stage Oocytes

**Figure 5:**
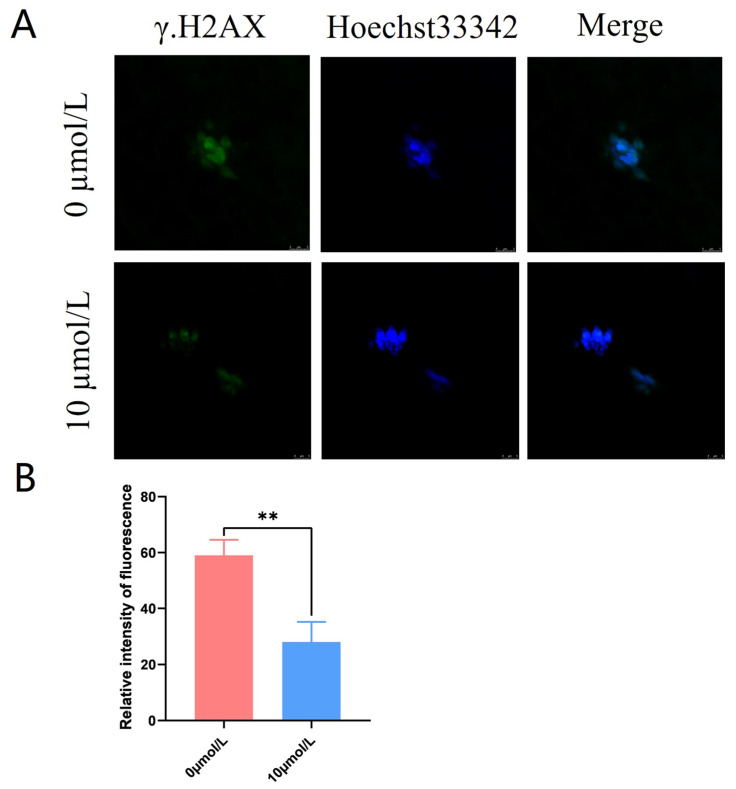
Effects of Catalpol on the DNA damage in porcine MII stage oocytes. (**A**) stained fluorescent picture with γ.H2AX; (**B**) statistics of staining fluorescence intensity with γ.H2AX. The mean ± SEM was used to represent all data. Asterisks indicate statistical significance (** *p* < 0.01). scale bar = 50 μm.

γ.H2AX was distributed in a star-shaped pattern on the chromosomes. The γ.H2AX fluorescence intensity was significantly lower in the 10 μmol/L catalpol treatment group compared to that in the control group (28.00 ± 7.21, n = 60, vs. 59.00 ± 5.57, n = 90, *p* < 0.01; Figure 5A,B).

### 3.7. Effects of Catalpol on Embryonic Development

**Figure 6:**
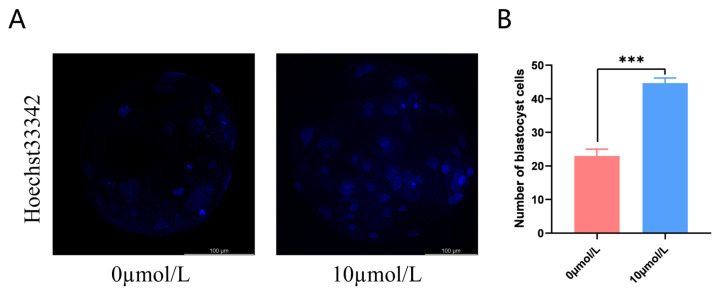
Effects of Catalpol on number of blastocyst. (**A**) stained fluorescent picture with Hoechst33342; (**B**) statistics of number of blastocyst cells. The mean ± SEM was used to represent all data. Asterisks indicate statistical significance (*** *p* < 0.001). scale bar = 100 μm.

After PA, cleavage rates in the 10 μmol/L catalpol treatment group were significantly higher than those in the control group. On day 7, the blastocyst formation rates were significantly higher in the 10 μmol/L catalpol treatment group than in the control group. (Table 3). The blastocyst cell numbers were higher in the 10 μmol/L catalpol treatment group compared to those in the control group (46.67 ± 1.53, n = 45, vs. 22.67 ± 2.08, n = 48, *p <* 0.001; Figure 6).

## 4. Discussion

IVM is a complex process regulated by multiple internal and external factors, and any change in this process can alter the quality of oocytes. Oocyte quality, in turn, affects subsequent embryonic development. Oxidative stress has the largest negative impact on oocyte quality during IVM, causing DNA damage, mitochondrial dysfunction, spindle abnormalities, and impaired embryonic cell development. Recent studies have shown that the addition of enzymatic or non-enzymatic antioxidants to in vitro culture systems effectively reduces oxidative stress damage and improves oocyte maturation and embryonic development [24,25].

Catalpol is a potent antioxidant, extracted from the Chinese herbal medicine *Dioscorea alata*, and has been shown to protect ARPE-19 cells from oxidative stress by activating the Keap1/Nrf2/ARE pathway [20]. In addition, catalpol protects primary cultures of cortical astrocytes from H_2_O_2_-induced toxicity by enhancing antioxidant enzymes to maintain GSH metabolic homeostasis [26]. Although catalpol is a strong antioxidant, its effects on IVM oocytes and subsequent embryonic development remains unclear. Buffalo and cattle oocytes require 24 h of IVM, while sheep oocytes require 22 h. Porcine oocytes mature slowly (42–46 h) and are more susceptible to oxidative stress [27,28,29]. Therefore, in this study, we investigated the effects of catalpol supplementation on COCs during porcine IVM.

CCs are essential for oocyte maturation [30,31]. CC expansion is positively correlated with oocyte developmental capacity; CCs provide nutrients to oocytes, exchange many molecular signals with oocytes, and communicate through gap junctions to regulate oocyte meiosis [32,33]. Previous studies have reported that the addition of antioxidant Nobiletin to bovine IVM medium can promote the production of E2 and P4 from CCs, thereby promoting nuclear and cytoplasm maturation in cattle [34]. Amphiregulin increased the developmental competence of pig oocyte-derived cloned embryos by increasing CC expansion and proliferation during IVM, according to Zhang et al. [35]. In the present study, the addition of catalpol to porcine oocyte IVM medium improved CC expansion by promoting HAS2, PTX3, and PTGS2 mRNA expression, thereby promoting nuclear and cytoplasmic maturation. These genes are related to CC expansion (an indicator of CC extracellular matrix synthesis). Generally, as the extracellular matrix structure is synthesized, CCs expand, inducing successful oocyte maturation before ovulation [36].

CGs are unique to oocytes, and their distribution affects normal meiotic progression. Their distribution has an important influence on oocyte biology [37]. Therefore, CG distribution is a marker of oocyte maturation; CGs in normal mature oocytes are distributed in a circular pattern in the cortical area [34,38,39]. Our findings showed that the rate of abnormal CG distribution was significantly lower in the 10 µmol/L catalpol treatment group. These findings suggest that catalpol promotes cytoplasmic maturation during porcine IVM. Our findings support previous studies by Kere et al. and Sovernigo et al., who found that antioxidants such as quercetin, cysteamine, carnitine, and resveratrol promoted bovine oocyte cytoplasmic maturation [25,40].

Mitochondria are key organelles for energy production and are the main sites of ROS production in cells [41]. They provide ATP for oocyte development and are essential for oocyte maturation, fertilization, and embryonic development [42,43]. The permeability of the mitochondrial membrane drives electron transport and ATP production. Mitochondrial membrane potential is the electrical potential difference between the inner membranes of mitochondria, depending on the respiratory chain, and is the source of mitochondrial oxidative phosphorylation and the formation of ATP [44]. Therefore, measuring membrane potential is the best method to evaluate mitochondrial function. In this study, MitoTracker fluorescent probes were used to study the effect of catalpol on mitochondrial function during porcine oocyte IVM. The addition of catalpol significantly enhanced oocyte mitochondrial function and improved oocyte maturation quality by increasing mitochondrial content. Catalpol improved membrane potential and increased mitochondrial content, which is consistent with a previous report that higher mitochondrial content is associated with higher oocyte quality [45].

Under improper environmental conditions, high ROS generation negatively impacts oocyte quality and embryonic development. Exogenous antioxidants protect oocytes from oxidative damage during IVM [46,47]. In this study, ROS levels in the cytoplasm of mature oocytes were significantly lower in the 10 µmol/L catalpol-treated group than those in the control group. Nie et al. discovered that antioxidants such as Mogroside V could reduce ROS levels in oocytes [45]; however, we discovered that antioxidants such as catalpol could alleviate oxidative stress in oocytes more effectively. Therefore, catalpol scavenged ROS and demonstrated antioxidant effects during IVM of porcine COCs.

Intracellular GSH production protects cells from oxidative stress and is essential for oocyte cytoplasmic maturation. [48,49]; 10 μmol/L catalpol increased mature oocyte cytoplasmic GSH levels. Several studies have shown that the expression of antioxidant factors (*CAT* and *SOD*) in oocytes is closely associated with ROS levels [50,51,52,53]. Lipids are the most complicated class of macromolecules among the myriad biological targets of oxidative stress. Lipid oxidation generates many byproducts, mainly aldehydes, which can exacerbate oxidative damage [54]. MDA is the most important and well-studied result of polyunsaturated fatty acid peroxidation. MDA levels in mature oocytes were considerably lower in the catalpol treatment group (10 mol/L) than in the control group. These results indicate that catalpol scavenges the MDA produced during the maturation of porcine oocytes. This further demonstrates the ability of catalpol to reduce ROS levels in oocytes and improve their maturation quality. Additionally, to track the mechanism underlying the antioxidative activity of catalpol, we measured the expression of antioxidant enzymes. Our study found that catalpol increased the transcriptional expression of the antioxidant enzymes *CAT*, *SOD1* and *GPX1* in oocytes. Therefore, we speculate that catalpol enhances oocyte quality by promoting the transcriptional levels of these genes in the porcine oocyte complex of the oocyte thalamus, reducing cytoplasmic ROS content, and enhancing the antioxidant capacity of oocytes.

Normally, high levels of ROS in cells inhibit the repair of DNA double-stranded breaks, triggering oxidative DNA damage, [55]. Therefore, we also examined the level of DNA damage in oocytes and found that the fluorescent signal was weak on the chromosomes of the treated group, whereas the control oocytes had obvious star-like spots on the chromosomes, indicating that catalpol treatment effectively reduced DNA damage in oocytes.

Oocyte quality is critical for embryonic development; however, embryonic development is also an important indicator of mature oocyte quality in vitro [45]. Catalpol supplementation during porcine oocyte IVM increased oogenesis and blastocyst formation in early embryos and had a catalytic influence on embryonic development according to this research. Catalpol also significantly increased the number of blastocysts. The positive effects of catalpol on mitochondrial activities, as well as decreases in intracellular ROS buildup, possibly explain this impact. Thus, we determined that 10 µmol/L supplementation of catalpol in the IVM medium improved the developmental competence of porcine oocytes after PA.

## 5. Conclusions

In conclusion, the supplementation of 10 μmol/L catalpol IVM increased CC expansion, as well as nuclear and cytoplasmic maturation. Catalpol supplementation improved DNA damage, COCs antioxidant capacity, and PA-derived porcine embryonic development. Therefore, catalpol is a potent antioxidant supplement for in vitro porcine oocyte maturation.

## Figures and Tables

**Table 1:** Quantitative real-time PCR primers of genes.

Genes	Primer Sequences	Length (bp)	Tm (°C)	Accession No.
*β*-actin	F: GATGACGATATTGCTGCGCTjayjayR: TTCTCCATGTCGTCCCAGTT	248	60	XM_021086047.1
*HAS2*	F: GAGTTTCTCTCCTCCTTGGAAjayjayR: ATGGGGGTTTCTAGAGATTTT	181	60	NM_214053.1
*PTX3*	F: GCTCTCTGGTCTGCAGTGTTGjayjayR: CGCATCTGGGAGTTCTCTAGC	179	60	NM_001244783.1
*PTGS2*	F: ATTTGTTGAATCATTTAGCjayjayR: CATTTCCTTCTCTCCTGTA	199	60	NM_214321.1
*SOD1*	F: GGGCACCATCTACTTCGAGCTjayjayR: CTCTCTTGATCCTTTGGCCCA	189	60	NM_001190422.1
*CAT*	F: AGATGAAGCATTGGAAGGAGC jayjayR: TCTCAGGAATTCTCTCCCGGT	183		NM_214301.2
*GPX1*	F: ACTACACCCAGATGAATGAGCjayjayR: ACACTTCTCGAAGAGCATGAA	182	60	NM_214407.1

**Table 2:** The effect of catalpol on porcine COCs first polar body (PB1) extrusion rate during IVM.

Catalpol Concentrations (µmol/L)	No. of Oocytes	Replicates	Rate of PB1 (%)
0	150	3	61.07 ± 2.80 ^c^
5	155	3	65.20 ± 1.82 ^ab^
10	145	3	72.33 ± 1.93 ^a^
50	149	3	66.93 ± 3.52 ^ab^
100	146	3	62.27 ± 2.11 ^bc^

Note: All data are expressed as the mean ± SEM. All experiments were replicated three times. a/b/c values in the same column with different superscripts significantly differ (*p* < 0.05).

**Table 3:** The effect of Catalpol supplementation during IVM on subsequent porcine embryo development.

Catalpol Concentrations (µmol/L)	No. of Embryos	No. of Cleavage (%)	No. of Blastocyst (%)
0	150	80 (52.67 ± 1.16 ^c^)	21 (13.99 ± 1.81 ^c^)
5	155	87 (56.15 ± 1.60 ^bc^)	26 (16.73 ± 0.97 ^c^)
10	145	96 (66.14 ± 2.80 ^a^)	37 (25.45 ± 1.81 ^a^)
50	149	88 (58.97 ± 2.13 ^b^)	29 (19.44 ± 1.38 ^b^)
100	146	78 (53.40 ± 1.52 ^bc^)	24 (16.40 ± 1.22 ^c^)

Note: All data are expressed as the mean ± SEM. All experiments were replicated three times. a/b/c values in the same column with different superscripts significantly differ (*p* < 0.05).

## Data Availability

No new data were created.
